# What have we learned about infant visual attention in the first 25 years of the 21st century?

**DOI:** 10.1016/j.infbeh.2025.102135

**Published:** 2025-09-07

**Authors:** Brianna K. Hunter, Erim Kızıldere, Shannon M. Klotz, Christian M. Nelson, Julie Markant, Lisa M. Oakes

**Affiliations:** a Center for Mind and Brain, University of California, Davis, USA; b Department of Psychology, University of California, Davis, USA; c Department of Psychology, Tulane University, USA

**Keywords:** Visual attention, Attention development, Eye-tracking, Egocentric views, Computational tools

## Abstract

At the beginning of the twenty-first century, the primary view of infant visual attention development focused on a transition across the first postnatal year from being stimulus-driven to goal-driven, reflecting a broader shift from subcortical to cortical control. This perspective was supported by decades of infant looking-time studies. However, our understanding of infant attention has significantly evolved over the past 25 years, shaped by both theoretical advancements and new technological and methodological tools. Researchers now understand that attention development reflects multiple interacting systems that have cascading effects across time. The availability of infant-suitable eye-tracking methods have allowed researchers to consider multiple aspects of attention by precisely measuring *when* and *where* infants look, emerging quantitative models of stimulus saliency and computational models of the visual system have deepened our understanding of bottom-up and top-down influences on infant attention, and new methods to evaluate infants’ egocentric views have allowed researchers to measure attention in naturalistic contexts. Thus, these innovations allowed us to address questions that were unthinkable 25 years ago. Here, we discuss how these advances have transformed our understanding of infant attention development and outline key directions for future research, paving the way for even more exciting discoveries in the next 25 years.

## 1. Introduction

Early philosophers emphasized the profound role of sensory experiences in shaping our understanding of the world, with Aristotle famously stating, “*perception is the first form of knowledge*.” This idea fueled a long line of scientific pursuits aimed to relate visual inputs, filtered by selective attention, to the acquisition of knowledge. *Visual attention*, or the ability to focus on one part of the visual environment while concurrently ignoring others, has cascading consequences for how people learn and interpret information (Posner, 1980; Treisman, 1964). It wasn’t until the late 1950s and early 1960s when researchers developed procedures to assess infants’ visual attention by measuring where and how long they looked, allowing us to draw conclusions about the perceptual and cognitive capabilities of infants (Fantz, 1956). It is important to point out that we now understand visual attention is not synonymous with looking; adult attention has often been studied in paradigms in which participants are explicitly instructed to not move their eyes (e.g., Posner cueing task; Posner, 1980). However, because infants cannot be given these verbal instructions, the study of visual attention in infancy has been dominated by measures of overt looking behaviors, and indeed, the terms “visual attention” and “looking” were used interchangeably in the first decades of research in this area (e.g., Cohen, 1973; Greenberg, 1977; Ruff & Turkewitz, 1975). The more recent introduction of physiological measures, including heart rate and EEG, has allowed researchers to ask questions about less observable aspects of attention as well, including engagement during sustained attention and covert attentional shifts (e.g., Colombo et al., 2001; Xie & Richards, 2016).

The primary conclusion drawn from the decades of research leading up to the end of the 20th century was that infants’ visual attention developed systematically across the first postnatal year. A large body of research focused on documenting and understanding the shift from reflexive to voluntary attention (e.g., Atkinson et al., 1996; Bronson, 1974, 1994), including how infants become able to inhibit reflexive attentional shifts (Atkinson et al., 1989; Johnson, 1995). This research also highlighted how very young infants’ attention is highly determined by bottom-up stimulus properties—such as high intensity contrast or salient movement—but increasingly with age, attention reflects top-down control based on higher-order factors, including familiarity and social significance (Colombo, 2001). These developments were attributed to a shift from subcortical to cortical processing (Bronson, 1974) as cortical regions involved in higher level cognitive functions gradually matured, such as those involved in goal-driven action and inhibition of prepotent responses (Johnson, 1990).

These initial conclusions were drawn primarily from studies in which human observers recorded infants’ looking times at one or two stimuli presented at a time (Fantz, 1956). These methods yielded valuable insights as they provide tight control over experimental stimuli. For instance, researchers explored how stimulus complexity, measured as the number of checks in a checkerboard, influenced infant attention while controlling for low-level perceptual factors (i.e., keeping the total number of black and white pixels constant across stimuli; Brennan et al., 1966). Although such methods reflected the technological limitations of the time, they laid a strong foundation for the field. Importantly, even as technological and theoretical advances have expanded the questions that can be addressed, researchers continue to rely on these fundamental methods for understanding the development of infant attention.

Over the first 25 years of the 21st century, our understanding of visual attention development has transformed due to significant technological and methodological advances in collecting and interpreting visual attention data from infants. Here, we discuss these advancements, highlighting the insights gained from these new findings. Our understanding of visual attention development has also become influenced by theoretical shifts in the field of developmental psychology that emphasize the role of interacting systems in the emergence and development of abilities (Oakes & Rakison, 2019; Smith & Thelen, 2003). As a result, researchers now consider attention development to reflect the interaction of multiple cognitive systems (Amso & Scerif, 2015; Amso & Tummeltshammer, 2020; Lynn et al., 2024; Reynolds & Romano, 2016) as well as the interaction between cognitive and other developing capacities (e.g., motor skills, perception) in the context of the whole child (Maia et al., 2024; Oakes, 2009, 2023a, 2023b; Smith & Gasser, 2005).

It is important to point out that much of the research on the development of visual attention has been largely conducted on infants from Western middle class families. According to sociocultural theory, children develop the ability to control their attention through their interactions with more experienced social partners within specific cultural and institutional contexts (e.g., Rogoff, 1990; Vygotsky, 1978). For instance, some cultures emphasize shared attention and guided exploration while others promote more independent attention (Bornstein et al., 1990; Bornstein et al., 1991). Although sociocultural theory has long shaped developmental thinking, there have been limited empirical comparisons of visual attention development across cultural contexts. This limited research suggests that basic attentional processes during infancy may indeed vary across cultures (e.g., DeBolt et al., 2025; Heise et al., 2025; Chavajay & Rogoff, 1999), socioeconomic statuses (e.g., Clearfield & Jedd, 2013), and language backgrounds (e.g., bilingual vs. monolingual; e.g., D’Souza et al., 2020; Singh et al., 2015). Thus conclusions from existing work must be considered in the cultural context in which they were observed, and assumptions about universality should be considered with caution.

## 2. Technological advances in the study of infant visual attention

In the last 25 years, a wide range of technological advancements has allowed for a much finer-grained understanding of the development of infant visual attention. For example, we now recognize multiple aspects of visual attention (e.g., overt versus covert orienting, sustained attention, disengagement) as a result of innovations in neuroimaging and physiological methods. This work often relies on multimethod approaches that combine behavioral measures of looking with neuroimaging measures, such as EEG (e.g., Pickron et al., 2018; Reynolds & Richards, 2005) or fNIRS (e.g., Emberson et al., 2017), as well as physiological measures such as heart rate deceleration (e.g., Colombo et al., 2001; Xie & Richards, 2016; Zhang et al., 2025). However, here we focus specifically on advances in the measurement and interpretation of infants’ looking behavior, building on the foundation laid by the decades of research beginning in the 1950s. In particular, we discuss how advances in 1) automated eye-tracking and 2) computational tools for quantifying stimulus properties have transformed the ways in which looking behaviors can be evaluated and related to specific stimulus properties (e.g., complexity, salience).

### 2.1. Automated eye-tracking

One of the most significant advances in the understanding of the development of visual attention was the introduction of automatic eye-tracking equipment and algorithms designed for collecting data from infants (Aslin & McMurray, 2004; Oakes, 2012). The types of manually coded data available prior to the 21st century (e.g., total looking time to a portion of a screen) became computable in a fraction of the time, facilitating research conducted using traditional looking time measures. These tools also enabled precise measurement of the spatial and temporal aspects of infants’ visual attention, revealing aspects of attention that traditional methods could not capture. Although it is still possible to aggregate looking times across space and time for analysis, new tools and models allowed researchers to leverage the nature of eye tracking data to answer new questions. Specifically, with these tools, we can now establish where *within* a stimulus infants look, allowing us to answer questions about how much time infants devote to specific facial features, such as the eyes and mouths of faces (Lewkowicz & Hansen-Tift, 2012; Oakes & Ellis, 2013). We can also present *more* stimuli simultaneously to infants, better reflecting the attentional demands infants experience as they explore the real world. For instance, we can measure infants’ attention to stimuli, such as faces, in visual search arrays in which multiple stimuli compete for attention at once (e.g., Gliga et al., 2009; Gluckman & Johnson, 2013; Hunter & Markant, 2021; Jakobsen et al., 2016; Kwon et al., 2016). Furthermore, whereas the prior methods emphasized sustained attention mechanisms (i.e., looking time), the temporal precision of eye trackers also yields insight into other aspects of attention, such as orienting (Leppänen, 2016). With measures of where infants looked every 1–33 ms, researchers can determine how quickly infants orient towards specific images. For instance, we can determine when infants shift their gaze to fixate on one of two simultaneously presented stimuli (e.g., Markant & Amso, 2016; Oakes & Kovack-Lesh, 2013; Tummeltshammer et al., 2014), providing insight into processes of attention orienting and attention disengagement. We can measure how quickly they move their eyes to a cued or uncued stimulus in the peripheral visual field (e.g., Ross-Sheehy et al., 2015), providing an understanding of covert attentional processes.

Further advances in eye-tracking, particularly mobile eye-tracking systems, have allowed researchers to examine visual attention in more naturalistic contexts, such as when infants are crawling or walking (Kretch et al., 2014), playing with a caregiver (Kızıldere et al., in press; Yu & Smith, 2013), or manually exploring objects (Chen et al., 2021). Importantly, these systems allow eye tracking from the infants’ own egocentric view, demonstrating how infants deploy attention during learning, in response to the behaviors of others, and as objects come in and out of view. This approach sheds light on both the factors that control infants’ attention in everyday life and the moment-to-moment fluctuations in attention during naturalistic interactions, offering insights that were previously inaccessible with traditional lab-based methods.

### 2.2. Tools for quantifying stimulus characteristics

Advances in computer vision and modeling during the first 25 years of the 21st century have provided complementary tools to analyze eye-tracking data, allowing for a more nuanced understanding of visual attention development. Specifically, developmental scientists have leveraged advances in computer and cognitive science to quantify image features and relate them to infant attention. This has advanced our knowledge by enabling a more systematic way to test established hypotheses. For example, whereas early work described the effect of physical salience on infant attention using simple visual patterns, such as checkerboards or objects presented in isolation (e.g., Brennan et al., 1966; Cohen et al., 1975; Fantz, 1956, 1963, 1964), we can now present infants with naturalistic scenes and use quantitative models of physical salience (i.e., *salience models*; Bylinskii et al., 2019; Judd et al., 2012) to more objectively measure low-level stimulus features of the scenes. Some of these salience models were inspired by the properties of V1 and V2 in primate visual cortex (e.g., Harel et al., 2007; Walther & Koch, 2006), allowing us to not only quantify salience in a wide range of displays but also to draw conclusions about the role of early visual cortex in infant looking behavior. The application of these tools has confirmed previous conclusions regarding the decreasing influence of physical salience on infants’ visual attention across the first postnatal year (Frank et al., 2009; Kwon et al., 2016; Pomaranski et al., 2021). Moreover, these tools have led researchers to recognize that the effect of salience is not an all-or-none factor; rather, it is a relative feature (DeBolt et al., 2023), composed of multiple low-level stimulus properties (Hunter et al., 2025), and must be considered within the context of the scene’s semantic features (Amso et al., 2014; Oakes et al., 2024).

The advent of these tools has also paved the way for both researchers of infant and adult attention to examine the influence of a variety of other low- and mid-level stimulus features, such as entropy, feature clutter, texture, and edge density (E. M. Anderson et al., 2024; Balas & Woods, 2014; McAdams et al., 2025; Rosenholtz et al., 2007). As a result, we are no longer limited to only considering whether infants attend to the most salient object in an isolated display. Instead, we also can examine how attention is influenced by a combination of features in more naturalistic contexts. Furthermore, researchers have used these tools to analyze the images taken from cameras mounted on infants’ foreheads (therefore capturing infants’ egocentric fields of view), providing insight into how the properties of scenes that infants actually see vary across development (E. M. Anderson et al., 2024).

New tools have also emerged that allow researchers to quantify higher-order properties of scenes that were once considered too complex to systematically measure. For instance, *meaning maps* can be created based on adults’ ratings of the semantic informativeness, or *meaning*, of isolated scene patches (Henderson & Hayes, 2017, 2018). Patches with uniform texture or no identifiable objects are typically rated as low meaning, and those with an identifiable object are often rated as high meaning. These ratings can be combined into a map that reflects the meaningfulness of each location within a naturalistic image. In general, adult fixations are better predicted by these meaning maps than by maps of physical salience (Hayes & Henderson, 2022; Henderson & Hayes, 2017; Peacock et al., 2023). We are just beginning to apply this approach to infants, but the initial findings suggest that infants’ fixations are also influenced by adult meaning ratings and that the effect of meaning increases across the first postnatal year (Oakes et al., 2024).

Finally, researchers have begun to use deep neural network models (DNNs) as tools for modeling how infants’ attention relates to low-, mid-, and high-level feature properties. The models are structured hierarchically, with a sequence of layers that extract increasingly abstract visual information from images, similar to how visual input is processed as it travels from V1 through the inferotemporal cortex in the visual system. In general, DNN layers reasonably approximate activity recorded from the sequence of areas along the adult ventral pathway (e.g., Cadieu et al., 2014; Güçlü & Van Gerven, 2015; Khaligh-Razavi & Kriegeskorte, 2014; Storrs et al., 2020; Wen et al., 2018; Yamins et al., 2014), which are notoriously challenging to measure among awake infant participants (though see Deen et al., 2017). Until higher-resolution fMRI from awake infants is more accessible and feasible, DNNs remain a valuable tool for exploring how different levels of visual abstraction guide attention in early development. For example, Kiat et al. (2022) related the spatial distribution of infants’ fixations to the pattern of activations across units in each layer within a DNN. They found that as infants matured, the spatial distribution of their fixations aligned more closely with the later layers of the DNN, suggesting that their attention was increasingly guided by higher-level visual representations.

## 3. The emergence of new theories of infant attention development

These innovations in technology and quantitative methods have allowed us to address questions that were unthinkable 25 years ago. These advancements have also reshaped how contemporary researchers studying adults view and discuss attention mechanisms. Specifically, researchers used to argue that adult attention selection is driven by either bottom-up or top-down factors (e.g., Corbetta & Shulman, 2002; Desimone & Duncan, 1995; Egeth & Yantis, 1997; Posner & Petersen, 1990). However, researchers have largely come to the agreement that this dichotomous view is overly simplistic (B. A. Anderson et al., 2021; Awh et al., 2012; Luck et al., 2021) and that these selection mechanisms may be difficult to clearly distinguish (B. A. Anderson, 2024). Recent theories propose that adult visual selective attention is guided by a priority map that integrates multiple factors—such as physical salience, prior learning, motivational relevance, and task goals—to dynamically compute the attentional priority of each location in the visual field (Todd & Manaligod, 2018).

One theoretical advancement in the field of visual attention development is that, similar to adults’, infants’ attention is not controlled by *either* bottom-up *or* top-down factors (Markant & Hunter, in press; Markant & Scott, 2018). Although the carefully designed experiments traditionally employed in infant research allowed us to examine bottom-up and top-down selection separately, it has become clear that these mechanisms may be functionally integrated and challenging to disentangle during more complex tasks—such as those afforded by eye-tracking methodologies—or in daily experiences. Thus, the findings of infant attention research over the past 25 years are consistent with the more nuanced theoretical framework adopted by adult attention researchers, indicating that a strict dichotomy between bottom-up versus top-down control cannot fully explain how infants attend. For example, Amso and Scerif (2015) proposed that bottom-up perceptual factors are inextricably linked to higher cognition; attention development occurs as a direct result of infants’ increased ability to process and integrate low-level stimulus information. Markant and Scott (2018) also proposed that biases in face recognition result from a dynamic interaction between bottom-up perceptual and top-down cognitive factors across the first postnatal year. Moreover, empirical work supports these claims: the effect of bottom-up perceptual factors on infants’ attention depends on *what* they are attending to. Hunter and Markant (2021) reported that when viewing arrays that contained pictures of objects (e.g., shoes, cups, bicycles) and a face, 6- and 11-month-old infants were more likely to look at the most physically salient distractor when the face in the array was an other-race face, compared to when the face in the array was an own-race face. Thus, infants’ attention to physically salient images was influenced by the top-down role of face identity. Similarly, Amso and colleagues (2014) investigated the ability of 4- to 24-month-old infants to detect faces within naturalistic scenes and found that older infants were more likely to detect faces that were more physically salient. In general, such research demonstrates that it may not be possible to understand the isolated roles of bottom-up or top-down factors in infants’ attention, but that researchers should consider how the two factors together determine infants’ attention.

The interaction between top-down and bottom-up attentional control can similarly be seen in how infants’ create their own views of the world through voluntary movements. In one study, 12-month-old infants used controlled head movements to maintain the distance between their head and an attended object, ensuring that the object was larger than surrounding items (Mendez et al., 2024). Thus, infants used top-down attentional control to manipulate the bottom-up physical salience of the objects in their field of view. The conclusion from this work is that the bottom-up versus top-down theoretical framework described by infant visual attention researchers at the beginning of the 21st century may not sufficiently explain the findings generated over the last 25 years. Instead, attention is a complex system, and reflects the interaction of multiple processes (Oakes, 2023b).

A second theoretical advance is the recognition that attention development reflects the cascading effects of multiple dynamically changing systems (Oakes 2023a, Oakes 2023b). Visual attention—just like other abilities such as short-term memory (Ross-Sheehy et al., 2003), motor skills (Malina, 2004), and perception (Kellman & Arterberry, 2000)—does not operate or develop in isolation. Although systems-based approaches to development have long emphasized the interconnectedness of developmental systems (Smith & Thelen, 2003; Thelen & Smith, 1994), such frameworks were not initially integrated into the study of visual attention development. Oakes (2009) argued that attempting to study abilities separately led us to the “Humpty Dumpty problem.” That is, we gained detailed insights into the development of individual processes with little understanding of how they interact in development.

A comprehensive understanding of visual attention development, therefore, requires examining it in the context of other developing systems, as infant vision evolves alongside changes in other domains. Motor milestones, in particular, provide infants with new opportunities to explore and shape their visual experiences. For example, studies equipping infants and toddlers with head-mounted cameras revealed that young infants’ visual experiences are initially dominated by their caregivers’ faces during close contact, such as cradling (Fausey et al., 2016; Jayaraman et al., 2015; Jayaraman & Smith, 2019; Sugden et al., 2014). However, by 9 months, infants spent relatively little time looking at their caregivers’ faces (Abney et al., 2020; Franchak et al., 2011; Suarez-Rivera et al., 2019; Yu & Smith, 2013, 2017). Instead, they focused more on caregivers’ hands and the objects they interacted with. These changes in the content of infants’ visual input reflect the development of postural control and locomotor abilities. Young infants with limited independent movement are typically held by their caregivers and therefore spend much of their time viewing their caregivers’ faces (Fausey et al., 2016). In prone and supine positions, their ability to visually explore their surroundings is restricted by posture and gravity (Adolph & Franchak, 2017). However, as infants develop motor skills, they gain greater control of their posture and head movement, expanding their visual field and creating more variety in their visual experiences (e.g., Kretch et al., 2014; Soska et al., 2010). Kretch and colleagues (2014), for example, demonstrated that infants’ field of view undergoes dramatic changes with the transition from crawling to walking. Importantly, infants’ developing visual attention does not simply reflect changes in motor development. Visual attention also develops alongside changes in infant-caregiver interactions (Karasik et al., 2011, 2014), social cognition (Mundy & Newell, 2007), and object recognition (Reynolds, 2015).

A third theoretical advance is the recognition that visual attention must be understood *in context*. That is, although significant understanding has been gained from the use of well controlled stimuli with low ecological validity, we may only fully understand the development of visual attention by expanding our understanding to the infants’ everyday lives. Advancements in technology and methods have led researchers to increasingly explore infants’ visual attention to naturalistic stimuli, such as photographs of everyday places and objects (i.e., naturalistic scenes; Oakes et al., 2024; Pomaranski et al., 2021; van Renswoude et al., 2019). Although scene viewing is not precisely the same as attending in a dynamic, 3-dimensional world, the study of infants’ attention to natural scenes has allowed us to make more general conclusions about how infants attend when presented with environments in which many visual stimuli compete for attention. For example, like adults, infants show a strong tendency to fixate on the center of the screen (van Renswoude et al., 2019) and tend to make more saccades across the horizontal axis (van Renswoude et al., 2016). These findings suggest that the biases in fundamental eye movement patterns observed in adults emerge early in development.

Studying attention in context is critical because many studies in the first 25 years of the 21st century demonstrate that visual input shapes the development of visual attention: everyday experiences influence attentional patterns. For example, 4-month-old infants with pets at home focused more on animals’ heads—an informationally rich feature—compared to those without pets (Hurley & Oakes, 2015). The amount of diversity in the faces of infants’ daily experiences influenced how they visually explored own- (familiar) and other- (novel) race faces (Ellis et al., 2017). Furthermore, as infants gain experience, they become increasingly able to control their visual input (Smith et al., 2018). For example, when infants between 18 and 24 months manually played with objects, their self-generated actions created many unique views of a single object which ultimately supported their object recognition and learning of the object names (James, Jones, Smith, et al., 2014; James, Jones, Swain, et al., 2014). Moreover, the limited research that has compared infant visual attention in different cultural contexts suggests that aspects of infants’ everyday lived experience may contribute to how attention develops (DeBolt et al., 2025; Heise et al., 2025).

## 4. Where do we go next?

The advances we have described thus far lead to the conclusion that understanding the development of visual attention requires more than considering a simple bottom-up and top-down dichotomy and that visual attention must be understood in the context of other developing processes as well as the environment. Infants’ visual attention at any given moment reflects many processes including arousal, perception, stimulus factors, and the infants’ history and goals. Understanding the development of this system requires considering how development in different domains interact. In particular, attention development reflects intertwining cascading changes (Oakes, 2023a), but the exact nature, developmental timescale, and variability across individuals in these changes are still uncharted. Measuring multiple developing systems within and across time in large, diverse samples will therefore allow for a better understanding of how the visual attention cascade unfolds. For instance, researchers may longitudinally measure how low-level stimulus features influence attention before and after infants achieve gross motor skill milestones (e.g., unsupported sitting, crawling, walking), providing insight into how the ability to actively control visual input through motor systems relates to infants’ ability to control their visual attention. Furthermore, infant attention researchers may measure infants’ perceptual capabilities, such as visual acuity or color discrimination thresholds, to directly relate perceptual and cognitive systems (see this approach in children, Lynn et al., 2024).

Furthermore, deeper understanding of infant visual attention will require that researchers measure attention in the real-world, as it dynamically interacts with other systems. This work should complement short-term experimental designs that allow for great control over confounding variables. For example, work by McAdams et al. (2025) found that, when presented with images of building facades, infants preferred looking towards the facades with greater edge orientation entropy. Complementary work by Anderson et al. (2024) indicated that infants’ egocentric views are dominated by simpler edge orientations. Together, this work suggests that McAdams et al.’s (2025) findings may reflect infants’ propensities towards novel stimuli. However, because stimulus features differ in their significance as a function of infants’ past experiences and knowledge, understanding the role of those features on infants’ visual attention requires studying them in context. It is important that future work continues to characterize the properties of infants’ visual inputs to better understand variability across individuals (for examples, see Anderson et al., 2024; Fausey et al., 2016; Kretch et al., 2014).

Finally, it is unclear how much of what we know about infant visual attention is indeed “universal”, and general to infants regardless of culture or type of visual experience, and how much reflects the specific experiences of the populations most commonly studied. One recent study revealed that whereas 6- to 9-month-old infants raised in Northern California were faster to fixate a target, 6- to 9-month-old infants raised in rural Malawi were more accurate in their fixations to the target (DeBolt et al., 2025). Another study found that East Asian 15-month-old infants tended to look longer at items in the background of a scene compared to 15-month-old North American infants (Heise et al., 2025). Differences are also observed in more naturalistic interactions. For example, Chavajay and Rogoff (1999) found that 14- to 20-month-old toddlers from a Guatemalan Mayan community were more likely to attend to multiple events at once, whereas White American toddlers were more likely to shift their attention back and forth between competing events, often focusing on just one event at a time. These studies demonstrate that a more complete understanding of the visual attention development requires including more diverse samples in our research.

To address these limitations in the field, we must increasingly incorporate: 1) longitudinal and multisystem approaches that track infants’ attention over time in the context of other developing skills (e.g., motor skills), social interactions, and environmental changes; 2) ecologically valid stimuli and measures that reflect how infants engage with their environment in natural settings; and finally, 3) samples that represent a broader range of diversity in experience.

Taking this approach will be facilitated by continuing to leverage the advancements in the field of computer science and computer vision to help analyze and interpret the data. We have only just begun to understand how advances in artificial intelligence and machine learning will yield even more promising and sophisticated tools to understand visual attention. For example, using transformers, a deep-learning architecture, DeepMeaning (Hayes & Henderson, in press; Hayes & Henderson, 2023) is a new automated method for identifying objects and semantic content within scenes. One limitation of this approach is that transformer models are trained on image-text relations, and thus the areas of images with the greatest semantic “meaning” identified by the model may not be the most relevant regions from a prelinguistic infant’s perspective. Nevertheless, these tools have the potential to help us move beyond surface-level scene statistics by representing the semantic relations between scene elements (Nuthmann & Henderson, 2010; Peacock et al., 2019).

Similarly, developments in computer vision have resulted in Grounding DINO, a transformer model that facilitates the detection of objects in scenes based on defined object or category labels (Liu et al., 2025). By integrating such tools with eye-tracking or head-mounted cameras, researchers can easily identify which objects infants commonly encounter in everyday interactions, determine the objects they fixate on and how their attention shifts over time, and analyze whether caregivers’ labels (e.g., “ball,” “rattle”) align with infants’ gaze towards objects. Furthermore, Human Pose Estimation (HPE) models have emerged as a computational tool for researchers to automate the analysis of large video datasets. For example, HPE can be used to automatically detect faces, hands, and other features in videos, such as those recorded from head-mounted cameras. Importantly, these models have shown great promise in their ability to replicate and extend well-established findings by characterizing infants’ nonverbal communication (Yurtsever & Eken, 2022) and quantifying how infants’ access to social information dynamically changes across development (Long et al., 2022). Critically, the ease and relative cost of applying these emerging machine-learning tools, compared to traditional behavioral coding methods, make them well-suited for application to datasets from understudied populations, providing greater diversity in our understanding of the relationship between culture and development (Long et al., 2022).

Finally, although infant researchers have begun to relate DNN models to infants’ looking patterns (Kiat et al., 2022), there are several uses of such models that will expand our knowledge. For example, researchers may examine how DNN models relate to different aspects of infants’ visual attention, providing insight into how visual abstraction relates to different components of infant attention (e.g., orienting vs. sustained attention). Furthermore, many existing DNNs do not account for the maturational state of the infant visual system and are not trained in ways that reflect how infants learn in their daily lives (Smith & Slone, 2017). Future models may incorporate biological constraints—such as limited acuity and immature high-level cortex—and be explicitly trained on the types of visual input infants experience. Such models may provide the best insight into the development of visual perception and attention.

## 5. Conclusion

Clearly, there have been significant shifts in our understanding of the development of visual attention in infancy in the first 25 years of the 21st century. Enabled by advances in technology, modeling, and an understanding of the adult attention system, our conceptualization of how visual attention develops during the first postnatal year has expanded from a relatively simple view to a more nuanced and sophisticated understanding that attention development is an unfolding cascade of events (Oakes, 2023a, 2023b; Oakes & Rakison, 2019). That is, attention development is a dynamic process, shaped by the whole child and their context. Built on the foundation established in the 20th century, we now understand that although the influence of physical salience on attention may decrease over infancy, it may actually operate in the context of priority maps, as has been described for adults (Luck et al., 2021; Todd & Manaligod, 2018). In addition, visual attention cannot be solely understood by examining infants’ looking as they view static, simple stimuli. We now recognize that we must also record infants’ eye gaze as they view complex stimuli (such as photographs and videos of everyday scenes), we must consider the infants’ own egocentric view, and we must study attention development in the context of other developing systems. By investigating attention in these contexts, which previously seemed impossible due to methodological limitations, we have gained important insights into the development of the attentional system, transforming our understanding of how infants perceive and learn from their worlds.

## Data Availability

No data was used for the research described in the article.
